# Attributable Costs of Postoperative Atrial Fibrillation among Patients Undergoing Cardiac Surgery

**DOI:** 10.1155/2018/3759238

**Published:** 2018-06-28

**Authors:** Pattamawan Kosuma, Sitichok Wachirasrisirikul, Arom Jedsadayanmata

**Affiliations:** ^1^Department of Pharmacy Practice, Naresuan University, Phitsanulok, Thailand; ^2^Department of Surgery, Buddhachinaraj Hospital, Phitsanulok, Thailand; ^3^Faculty of Pharmacy, Thammasat University, Pathum Thani, Thailand

## Abstract

**Background:**

Postoperative atrial fibrillation (POAF) is the most common complication among patients undergoing cardiac surgery. However, data on the economic burden and resource utilization associated with POAF in Asian population are limited. The present study aimed at estimating medical costs attributable to POAF after cardiac surgery in Thai population.

**Methods:**

We analysed data from claims database of patients who underwent valve replacement, coronary artery bypass grafting (CABG), or a combination of both procedures at a tertiary-care, academic hospital in Thailand. Multiple linear regressions of log-transformed costs were developed with the occurrence of POAF and preoperative patient characteristics as covariates. After back-transformation to the original scale, costs attributable to POAF were estimated from the mean difference between patients with and without POAF.

**Results:**

Of 711 patients undergoing cardiac surgery, 241 (30.94%) developed POAF over a median hospitalization of 10 days. Patients with POAF utilized more resources than those without POAF. POAF was an independent predictor and increased cost by 23% in linear regression model. On average, patients with POAF had higher medical costs than those without POAF (269,000 versus 218,999 Thai Baht (THB)) with a mean difference of 50,000 THB (1,667 USD). The difference was observed in patients undergoing isolated valve surgery (47,761 THB (1,592 USD), 95% CI: 39,809–55,712), CABG (50,865 THB (1,696 USD), 95% CI: 37,233–64,496), and a combination of both procedures (72,287 THB (2,410 USD), 95% CI: 49,910–94,405).

**Conclusions:**

In a single-institution study in Thailand, POAF is associated with increased resource use and medical costs among patients undergoing cardiac surgery. Effective strategies to prevent POAF should be implemented to reduce its economic burden.

## 1. Introduction

Postoperative atrial fibrillation (POAF) is the most common complication among patients undergoing cardiac surgery [1]. The incidences of POAF have been reported from 10 to 60% depending on the study population and POAF definition [2, 3]. POAF usually occurs within the first 2-3 days after surgery and is associated with other postoperative complications including ischemic stroke, heart failure, and acute kidney injury [2, 3]. Standard therapy for POAF, namely, antiarrhythmic agents and anticoagulants, could cause unwanted effects by inducing proarrhythmia and bleeding [3]. As a result, POAF may complicate the course of hospitalization, compromise clinical outcomes, prolong hospitalization, and increase costs of care among cardiac surgery patients [4–9].

Despite its potential impact on morbidity and resource utilization, few studies evaluated the economic burden of POAF in Asian population. Previous reports were conducted mostly in Western countries [4–10]. Such economic information might not be easily transferable across countries because the cost of care and resource utilization could vary among regions, depending on practice pattern and resource available. Knowing the economic burden of POAF is important to determine the impact of interventions to prevent this complication in the context of Asian health services. Thus, the present study aimed at estimating direct medical costs attributable to POAF in Thai population undergoing cardiac surgery. The findings will have significant implications for policy making.

## 2. Methods

### 2.1. Data Collection

We retrospectively analysed a cohort of patients who underwent valve replacement or coronary artery bypass grafting (CABG) or a combination of both procedures at a 1000-bed, tertiary-care, academic hospital in Thailand. All data were retrieved electronically from claims database at the study hospital. The claims database provided information on patient demographics, a principle diagnosis, types of surgery performed, comorbid conditions prior to admission, complications during hospitalization, discharge disposition, individual service charges, and pharmacy product utilization.

Patients were eligible for recruitment if they were 18 years of age or older, had undergone CABG identified with the International Classification of Diseases, Ninth Revision (ICD-9) coding system (ICD-9: 36.10 to 36.16), and/or had undergone valve surgery (ICD-9: 35.20 to 35.28) between January 1, 2005, and June 30, 2012. Patients were excluded if they died within 24 hours of surgery.

The occurrence of POAF was identified with the International Classification of Diseases, Tenth Revision (ICD-10) code for AF (I48) when it was recorded as a complication during hospitalization. Because AF occurring prior to admission will be recorded under comorbid conditions, this enabled us to distinguish POAF from AF occurring prior to cardiac surgery.

The claims database provided data on inpatient charges from individual services during hospitalization. A unique patient identifier was used to combine datasets, enabling estimation of total charges for each patient. Charges were converted to costs with cost-to-charge ratios reported specifically in each year by the hospital accounting department. We did not adjust medical costs for inflation over the study period.

### 2.2. Data Analysis

Baseline characteristics between patients with and without POAF were compared using chi-square for categorical variables and the independent *t*-test or Mann–Whitney *U* test as appropriate for numerical variables.

The total medical costs in Thai Baht (THB) were separated into cost components according to the services provided including supplies and devices; room, board, and nursing; pharmacy products; laboratory and testing; operations; and other services. We compared these unadjusted cost components between patients with and without POAF using mean difference and independent *t*-test. For intercountry comparisons, costs in THB can be converted into United States Dollar (USD) using the exchange rate of ≈30 THB for 1 USD [11].

The primary aim of the study was to determine medical costs attributable to POAF among patients undergoing cardiac surgery. We first performed simple linear regression of log-transformed costs with preoperative patient characteristics as independent variables to identify potential confounders. Potential confounders were defined as those variables associated with the log-transformed cost with a *p* value less than 0.2 on univariable analysis. The occurrence of POAF together with all potential confounders was forced to enter the multiple linear regression model, regardless of their statistically significant contribution to the final model. The heteroscedasticity of the regression residuals on the log scale was examined with White's test [12], and no significant heteroscedasticity was observed. Thus, the adjusted costs were retransformed back to their original scales with correction of retransformation bias using Duan's smearing estimator [13]. The attributable cost of POAF was determined by the mean difference of adjusted costs between patients with and without POAF.

Statistical analyses were performed using the Stata software. Unless otherwise indicated, the tested results were considered statistically significant with a *p* value < 0.05.

### 2.3. Ethical Issues

The study protocol was approved by the Institutional Review Board on Human Research at Naresuan University and the Human Research Committee at the study hospital prior to data collection.

## 3. Results

### 3.1. Patient Characteristics

The cohort consisted of 711 patients undergoing cardiac surgery during the study period. As shown in Table 1, the mean age was 56.07 ± 12.58 years and more than half of the cohort were male. Hypertension was the most prevalent (32.63%) comorbid condition. The majority (68.50%) underwent isolated valve surgery, while 24.61% and 6.89% underwent isolated CABG and concomitant CABG with valve surgery, respectively.

Overall, POAF developed in 220 patients (30.94%) during a median length of stay (LOS) of 10 days (interquartile range 3–17 days). Compared to those without POAF, patients with POAF were older (57.93 ± 12.00 versus 55.24 ± 12.58; *p*=0.008) and had fewer diagnoses of atrial fibrillation prior to operation (9.09% versus 15.07%; *p*=0.03), but more histories of chronic obstructive pulmonary disease, diabetes mellitus, dyslipidemia, hypertension, and ischemic heart diseases.

### 3.2. Resource Utilization

Patients with POAF spent 1 more day in hospital compared to those without POAF (median LOS, 11 days versus 10 days; *p* < 0.01). The resource utilization among patients with and without POAF was compared, as shown in Table 2. On average, patients with POAF incurred higher unadjusted costs during hospitalization in every cost component (*p* < 0.001). The greatest difference was observed in costs associated with utilization of pharmacy products (19,210 THB (640 USD)) accounting for 35% of the total mean difference.

### 3.3. The Attributable Cost of POAF

We adjusted the effects of potential confounders on the association between medical costs and the occurrence of POAF by developing a multivariable linear regression model. Because costs were heavily right skewed, we log-transformed the total costs prior to regression modelling.  Table 3 shows that, after adjusting for other variables, POAF still contributed significantly to the total cost (*β* = 0.21; 95% CI 0.15–0.27; *p* < 0.001). In other words, a patient undergoing cardiac surgery who developed POAF had 23% higher medical cost during hospitalization than a patient who did not develop POAF.

Table 4 shows that an occurrence of POAF incurred higher costs to patients undergoing either isolated valve replacement, CABG, or a combined procedure. The highest attributable cost was observed in patients undergoing a combined procedure (72,287 THB (2,410 USD) per patient).

## 4. Discussion

The results of the present study demonstrate that POAF is associated with higher medical cost independently of preoperative characteristics among patients undergoing cardiac surgery. The findings underscore a significant contribution of POAF to economic burden among patients who undergo cardiac surgery. To illustrate, about 10,000 patients undergo cardiac surgery in Thailand each year, and one-third of these patients will develop POAF. Given that POAF increases medical cost by 50,000 THB (1,667 USD) per patient according to our study, this will incur an additional cost of 170 million THB (5.7 million USD) annually. Thus, an effective intervention to prevent POAF or mitigate its severity if implemented would result in a significant reduction of health-care expenditure for the country.

The evidence that POAF prevention may save the cost of care in cardiac surgery can also be inferred from the cost-effectiveness study of an intervention to prevent POAF. For example, Gillespie et al. [5] reported that use of beta-blockers for prophylaxis of POAF in cardiac surgery was associated with a significant reduction in the incidence of POAF, hospital LOS, and total hospital cost. The results of our study agree with this finding and further emphasize beneficial effects of POAF prevention in cardiac surgery to Asian population.

Our data indicate that POAF was associated with more resource use from all service departments. In support of this observation, our results concurred with those reported by Hravnak et al., who found that POAF in patients undergoing isolated CABG resulted in more resource utilization, including laboratory test and pharmacy products [4]. The incremental cost was greatest in room services accounting for 26% of the total difference [4]. In our study, the major resource utilization driving the incremental cost of POAF was the pharmacy product utilization accounting for 35% of the total difference. This disparity is possibly explained by a variation in how service costs are established between hospitals, further emphasizing the confined application of economic data among various institutions and geographic locations. More importantly, both studies provided consistent evidence that the POAF incurs higher resource utilization in cardiac surgery patients.

We observed that patients with POAF stayed in hospital about 1 day longer than those without POAF. The difference in LOS would not totally explain the higher costs associated with POAF in this study. Consistent with our findings, previous studies reported that the LOS differed by approximately 1 day despite much higher costs observed among patients with POAF [4, 6]. Other studies reported a longer incremental LOS, although the results varied widely from 3 to 8 days [5,7–10]. In a more recent study, the occurrence of POAF after a combined CABG and aortic valve replacement increased hospital LOS by 3 days with an incremental cost of 9,000 USD [14]. This discrepancy in LOS possibly reflects the difference in study population, practice pattern among institutions, and study designs. Despite the difference in LOS, all studies consistently observed an increase in medical costs associated with POAF [4–10].

Although the cost attributable to POAF was observed in all types of cardiac surgery, the highest incremental cost was associated with the combined procedure. Our results agree with those reported by Mahoney et al. [14], who found that the incremental cost associated with POAF was highest among patients undergoing a combined procedure. Additionally, LaPar et al. [15] reported that patients who underwent combined CABG and valve replacement accrued higher costs and longer LOS than did those who had an isolated procedure. Patients with combined cardiac surgery had higher rates of overall complication, and this may drive incremental cost of combined cardiac surgery [15]. We postulated that a greater incremental cost of POAF in patients undergoing a combined procedure was possibly due to more frequent complication related to POAF. From our data, medical costs were not much different among patients who underwent an isolated or a combined procedure and did not develop POAF (Table 4), suggesting that POAF itself likely incurred higher costs after a combined procedure. However, we cannot exclude the possibility of data imprecision due to a smaller number of patients undergoing a combined procedure. This finding should be further explored to gain additional insights on the economic burden of POAF in relation to distinct types of cardiac surgery and the impact of its prevention.

Some limitations of the present study merit discussion. First, this study utilized a cohort of patients to determine the attributable cost of POAF. Due to its retrospective and observational nature, the present study was subject to several factors confounding the association between cost and POAF. We tried to alleviate this problem by developing a regression model to adjust for preoperative characteristics; however, we acknowledge the presence of other unmeasured confounders. Previous studies revealed that preoperative together with intraoperative and postoperative characteristics explained variation in total costs of cardiac surgery better than preoperative factors alone [16–18]. Thus, the estimated cost in this study may not be totally adjusted for. Of note, most of the previous studies estimated the attributable cost of POAF by determining the difference in unadjusted costs between patients with and without POAF [4–8]. Such method would also subject to several potential confounders due to difference in baseline characteristics and disease severity of the participants. Second, the present study utilized secondary database as a data source, this may raise the question of result validity. Misclassification of POAF could have occurred if the POAF was perceived as insignificant and was not recorded into patient charts by care providers. The lack of ECG records from claims database prevented us from verifying the occurrence of POAF. This could lead to an underestimation of POAF incidence and false estimation of the POAF cost. Last, this study was conducted with data from a single institution, and this may limit generalizability of the result to other settings. However, the incidence of POAF and its effect on resource utilization observed in our study were well consistent with other studies [4–9]. We therefore believe that the results likely provide a valid and generalizable estimate of the attributable cost of POAF and could be used in assessing the cost-effectiveness of interventions to prevent an occurrence of POAF among patients undergoing cardiac surgery.

## 5. Conclusions

In a single-centre study in Thai population, POAF is consistently associated with an increase in medical costs among patients undergoing valve surgery, CABG, or the combination. Effective strategies to prevent POAF should be implemented to reduce its economic burden.

## Figures and Tables

**Table 1:** Preoperative characteristics of patients in the study.

Characteristics	All patients (*N*=711) *n* (%)	With POAF (*N*=220) *n* (%)	Without POAF (*N*=491) *n* (%)	*p* value^*∗*^
Age (mean ± SD) (years)	56.07 ± 12.58	57.93 ± 12.00	55.24 ± 12.58	0.008
Male	402 (56.5)	135 (61.4)	267 (54.4)	0.080
*Comorbid conditions*				
Cerebrovascular disease	2 (0.3)	1 (0.5)	1 (0.2)	0.560
Chronic kidney disease	23 (3.2)	11 (5.0)	12 (2.4)	0.075
Congestive heart failure	94 (13.2)	33 (15.0)	61 (12.4)	0.340
COPD	23 (3.2)	12 (5.5)	11 (2.2)	0.025
Diabetes mellitus	107 (15.1)	50 (22.7)	57 (11.6)	<0.001
Dyslipidemia	192 (27.0)	75 (34.1)	117 (23.8)	0.004
Hypertension	232 (32.6)	92 (41.8)	140 (28.5)	<0.001
Ischemic heart disease	108 (15.2)	47 (21.4)	61 (12.4)	0.002
Preoperative atrial fibrillation	94 (13.2)	20 (9.1)	74 (15.1)	0.030
*Types of surgery*				0.332
Isolated valve replacement	487 (68.5)	144 (65.5)	343 (69.9)	
Isolated CABG	175 (24.6)	62 (28.2)	113 (23.0)	
Valve replacement + CABG	49 (6.9)	14 (6.4)	35 (7.1)	

^*∗*^Between patients with and without POAF using chi-square, except for age which was tested for the difference by independent *t*-test. COPD, chronic obstructive pulmonary disease; CABG, coronary artery bypass graft; POAF, postoperative atrial fibrillation.

**Table 2:** Medical costs according to service components in patients with and without POAF.

Services	With POAF (THB)	Without POAF (THB)	Mean difference (THB)	*p* value^*∗*^
Pharmacy	43,328	24,118	19,210	<0.001
Supplies and devices	103,933	90,628	13,304	<0.001
Room, board, and nursing	26,771	18,593	8,178	<0.001
Laboratory and testing	23,948	17,709	6,238	<0.001
Operations	56,062	50,309	5,753	<0.001
Others	20,571	15,861	4,709	<0.001
Total cost	274,613	217,219	54,393	<0.001

^*∗*^Independent *t*-test. POAF, postoperative atrial fibrillation; THB, Thai Baht.

**Table 3:** Predictors of total costs in multivariable linear regression model.

Variables	*β*	95% CI	*p* value
Postoperative atrial fibrillation	0.21	0.15–0.27	<0.001
Age > 60 years	−0.01	(−0.06)–0.05	0.823
Male sex	−0.01	(−0.06)–0.04	0.731
Preoperative atrial fibrillation	0.08	(−0.00)–0.15	0.057
Chronic obstructive pulmonary disease	0.14	(−0.00)–0.29	0.058
Ischemic heart disease	0.15	0.07–0.22	<0.001
Congestive heart failure	0.21	0.13–0.29	<0.001
Chronic kidney disease	0.26	0.11–0.41	0.001
Dyslipidemia	−0.07	(−0.13)–(−0.01)	0.027

**Table 4:** Attributable costs of POAF according to types of surgery.

Types of surgery	With POAF (THB)	Without POAF (THB)	Mean difference (THB)	95% CI	*p* value^*∗*^
Isolated valve replacement	268,740	220,979	47,761	39,809–55,712	<0.001
Isolated CABG	266,500	215,635	50,865	37,233–64,496	<0.001
Combined valve replacement and CABG	282,747	210,460	72,287	49,911–94,406	<0.001
All patients	269,000	218,999	50,001	43,440–56,561	<0.001

^*∗*^Independent *t*-test. CABG, coronary artery bypass graft; POAF, postoperative atrial fibrillation; THB, Thai Baht.
